# Childhood Adversities and Adult Headache in Poland and Germany

**DOI:** 10.1371/journal.pone.0148162

**Published:** 2016-02-09

**Authors:** Bettina Reuchlein, Lea Henn, Tamara Brian, Katarzyna Schier, Jochen Hardt

**Affiliations:** 1 Medizinische Psychologie und Medizinische Soziologie, Klinik für Psychosomatische Medizin und Psychotherapie, Universitätsmedizin der Johannes Gutenberg-Universität Mainz, Mainz Germany; 2 Faculty of Psychology, University of Warsaw, Warsaw, Poland; Taipei Veterans General Hospital, TAIWAN

## Abstract

**Objective:**

Various childhood adversities have been found to be associated with chronic pain in adulthood. However, associations were moderate in most studies, i.e. odds ratios (OR) were between one and two.

**Method:**

An internet survey was performed in 508 Polish and 500 German subjects. A total of 19 childhood adversities were selected and their associations with headaches explored. Age, gender and country were included as potential confounders, as well as their two-way interaction with the risk factors.

**Results:**

Two strong risk factors were identified. (1) A combined score for physical and emotional neglect showed an odds ratio (OR) of 2.78 (p < .002) to the frequency of headache in adulthood as a main effect. (2) Father having had chronic pain showed an OR of 4.36 (p < .001) with headache in adulthood for women, but not for men (OR = 0.86, p < .556). The majority of the examined childhood adversities were not associated with adult headache, neither when tested individually nor as a sum score.

**Conclusion:**

This study confirms results from previous ones that childhood adversities may play a role in the development of adult headache, but it is a rather minor one. Contrary to other studies, neglect turned out to be one of the strongest predictors.

## Introduction

In developed countries, chronic pain is one of the most challenging tasks health care systems face) (e.g. [1]). It is frequent in the populations of these countries and difficult to treat) (e.g. [2]). Estimates of prevalences of chronic headaches are strongly dependent on the underlying definitions of headaches [3]. Breivig et al. [4] used a definition comprising four criteria: (1) pain severity ≥ 5 on the numeric scale between 0 and 10, (2) duration longer than six month, (3) frequency usually at least one attack per week, one attack in the last month. Based on a survey over 15 European countries comprising almost 50,000 respondents, 19% met these criteria. A review of reviews from 1999–1012 comprising over 300 000 subjects all over the world came to a similar result, i.e. a prevalence of 22% [5]. Low income countries seems to have somewhat lower prevalences, however, cities in India like Colcatta or Chennai also lie at 21% and 22% [6]. Satisfaction with health care services in patients with chronic pain is low [4], communication with doctors often difficult for the patients [7]. Most patients with severe headaches use analgesics, but there is evidence that a substantial amount of patients does not achieve satisfactory pain relief through their treatments [8]. Multidisciplinary treatment has proven to be better for most pain patients, but is rarely applied [9]. Very frequent headaches of ≥ 15 days per months were estimated in Denmark for 3.3% of the population with half of them having concurrent medication overuse [10].

Childhood adversities have been associated with a wide range of psychological and somatic symptoms in adulthood and adolescence. Engel [11] suggested that negative physical or emotional experiences, especially physical and sexual abuse in childhood, lead to the development of chronic pain. Various studies show associations between physical symptoms, particularly pain, and childhood adversities (e.g. [12, 13]). Scott et al. [14] examined eleven childhood adversities in a sample of 18.000 adults from 10 countries. For eight out of the eleven, they found significant associations with frequent headache. However, in this study the associations were generally not strong, ORs ranged only between 1.2 and 1.7. A meta-analysis of 16 retrospective studies identified relationships of similar magnitude [13].

In sum, it seems likely that childhood adversities constitute a risk factor for pain development in adulthood—even though the possibility of bias in retrospective studies cannot be ruled out to date. The aim of the present study is to explore the associations of 21 childhood risk factors with adult headaches in a Polish and a German sample.

## Methods

### Sample

Subjects were asked to fill out an online questionnaire on the platform of a commercial company which usually performs market research (http://www.linequest.de). The survey contained about 280 items. It comprised 508 Polish and 500 German participants who received a compensation of about € 4,30. The sample size calculation was based on a mean difference of d ≥ .25 [15] between the Polish and German sample to be detected at alpha = .01 with a power of .90 (sampsi: [16]). Participants gave informed consent to their contribution to research on mental health in combination with various circumstances of life in Poland and Germany. Data collections were performed in summer 2008, it took about a week in each country. The scientific background of the study was posted on the homepage of the University of Mainz during data collection. The ethics commissions of the Landesärtztekammer Rheinland-Pfalz (Nr. 837.185.07) and the University of Duesseldorf (5720) approved the project.

In Poland, the mean age of the subjects was about 39 years, and slightly more than half were female. In Germany, a gender and age stratified sample was drawn. Most had a spouse or partner. In Poland, more participants were in partnerships than in Germany. Supplementary sample characteristics are displayed in Table 1.

**Table 1:** Sample description.

	Poland	Germany	
N	508	500	Test for differences
Gender: % female	56.3	50.0	*χ*²_(1)_ = 4.02, p < .045
*Age*: mean *(sd)*	38.7 (14.4)	44.8 (16.1)	t_(1006)_ = 6.40, p < .001
**Familiy status (%)**			
Married	48.8	43.0	
Partnership > 6 months	23.8	23.2	
Partnership < 6 months	3.5	3.8	
No partnership	17.3	26.6	
Other	6.5	3.4	*χ*²_(4)_ = 16.7, p < .002
**Headaches (%)**			
Never / rarely	45.3	50.6	
Sometimes	35.2	32.4	
Often / very often	19.5	17.0	*χ*²_(2)_ = 2.9, p < .229

### Variables

Participants were asked how often they had headaches: never, seldom, sometimes, often, or very often. The first two and the last two categories were combined due to small counts in the extremes leading to a variable with three categories.

Five items concerning negative personal experiences were assessed: childhood sexual abuse was coded “1” when a subject reported any unwanted sexual experience with someone at least five years older before the age of 15 and “0” otherwise. Harsh physical punishment was coded “1” if the subject was regularly beaten, coded “0.5” for often and “0” otherwise. Physical abuse was defined as having been beaten so hard that bruises occurred. It was coded in four steps according to how often it occurred. Threats of physical violence meant that there was someone in the family the subject was afraid of and was coded like physical abuse. For the latter two variables, “0” meant never, “0.33” once, “0.66” sometimes (2–10 times) and “1” often (i.e. more than 10 times). Neglect was assessed utilizing a ten-item score comprising items such as “I had to wear dirty clothes” or “there was always someone who cared for me” (reversed). The scale has a Cronbach’s alpha of .82 in Poland and .88 in Germany and was described in detail by Brian et al. [17].

Six questions on family adversities were derived from the “Mainz Structured Biographical Interview” [18] and included: parental separation or divorce before age 15, violence between parents defined as regular physical arguments. Family discord was a score of five topics (temperament, alcohol, finances jealousy and other) where parents might have had conflicts. Economic hardship in the family was assessed for two separate times, ages 0–7 and 7–14, and combined to a score, in which higher values stood for more economic hardship. Being a planned child had two categories (probably yes vs. don’t know / probably not). Did not grow up with natural parents was coded yes vs. no.

Furthermore, we assessed various parental disorders that may be connected with the development of headaches: mother or father physically ill, chronic pain, mental problems and alcohol abuse. All were coded “0” if absent and “1” if present. We did not include questions about drugs other than alcohol because such prevalences were very low in Poland and Germany. In the present study, all childhood adversities were recoded into values between “0” and “1” to allow comparisons of the odds ratios. In Table 2, binary variables are reported as percentages, and continuous variables with mean and standard deviation.

**Table 2:** Prevalences of childhood adversities.

Childhood adversity	%	Mean	SD
Negative **personal experiences**			
Sexual abuse	3.0		
Harsh physical punishment^1^		0.18	0.29
Physical abuse		0.13	0.27
Threat of physical violence		0.12	0.28
Neglect: Score of ten items		0.21	0.18
**Family adversities**			
Parental separation/divorce before age 15	20.4		
Violence between parents	18.2		
Family discord		0.79	0.24
Family economic adversity		0.48	0.26
Not always with natural parents	20.1		
Not being a planned child	25.3		
**Parental disorders**			
Mother physically ill	8.4		
Father physically ill	8.4		
Mother any chronic pain	11.5		
Father any chronic pain	7.7		
Mother mental problems	10.9		
Father mental problems	6.5		
Mother alcohol problem	4.1		
Father alcohol problem	18.8		

^1^ this variable had 58 “don’t knows” recoded into missing values.

Childhood adversities were assessed similarly but not identically to the Adverse Childhood Experiences International Questionnaire [19]. The main differences were that the WHO uses the first 18 years as a time frame, while we used the first 14 years. Additionally, neglect was assessed in more detail, here. The analyses follow basically the WHO binary analyses scheme.

### Statistical Analysis

Beside bivariate associations as displayed in Table 3, an ordered logistic regression analysis was performed for each risk factor (Table 4), since there is a single response with three categories in this study. Age, gender and country were added as potential confounders, as well as their two-way interactions with any risk factor. A backward selection of significance tests was performed: first the interaction terms were removed if non-significant, then the main effects. The risk factor was always kept in the model until the end. The alpha level for all statistical tests was set to .01 (two-tailed), accordingly 99% confidence intervals are reported in Table 3. Apart from the variable “harsh physical punishment”, which contains 58 “don’t know” values, which were interpreted as missing data, there were no missing items in the internet survey. The survey programme prompted the respondent for an answer when an item was left open. Calculations were performed using STATA 12 [16]. We report the ORs for the association between risk factor and headache. If no interaction effect became significant, a single OR was reported, if any interaction term became significant, separate ORs for subgroups are displayed in Table 4.

**Table 3:** Bivariate associations between headache and childhood risk factors.

Childhood adversity	Women OR 99% CI			Men OR 99% CI		
**Negative personal experiences**						
Sexual abuse	0.96	0.35	2.65	0.79	0.09	7.00
Harsh physical punishment	0.91	0.43	1.93	1.13	0.48	2.68
Physical abuse	0.92	0.41	2.07	1.36	0.58	3.20
Threat of physical violence	0.98	0.46	2.07	1.69	0.71	3.98
Neglect	1.47	0.51	4.02	6.09	1.47	25.32
**Family adversities**						
Parental separation	1.12	0.68	1.84	1.71	0.94	3.11
Violence in family	1.27	0.77	2.10	1.53	0.79	2.96
Family discord	0.54	0.24	1.23	0.49	0.17	1.36
Family economic adversity	0.93	0.42	2.05	0.97	0.37	2.56
Not always with natural parents	0.64	0.38	1.07	1.47	0.79	2.73
Not being a planned child	0.93	0.59	1.47	1.32	0.74	2.35
**Parental disorders**						
Mother physically ill	0.72	0.35	1.45	1.37	0.58	3.62
Father physically ill	1.84	0.85	3.95	0.90	0.38	2.15
Mother any chronic pain	0.81	0.44	1.51	1.91	0.90	4.08
Father any chronic pain	3.55	1.51	8.31	0.81	0.32	2.04
Mother mental problems	0.88	0.47	1.63	2.26	1.01	5.06
Father mental problems	1.46	0.66	3.20	1.04	0.39	2.82
Mother alcohol problem	0.84	0.34	2.10	2.25	0.61	8.27
Father alcohol problem	1.41	0.86	2.34	1.34	0.71	2.55

**Table 4:** Adjusted associations between headache and childhood risk factors.

Childhood adversity	Odds Ratio Women Men			p_main_	p_int._^1^
**Negative personal experiences**					
Sexual abuse		0.97		.934	.824
Harsh physical punishment		1.08		.728	.587
Physical abuse		1.14		.560	.369
Threat of physical violence		1.22		.362	.191
Neglect		2.78		.002	.042
**Family adversities**					
Parental separation		1.32		.068	.190
Violence in family		1.31		.081	.661
Family discord		1.29		.046	.863
Family economic adversity		1.39		.204	.856
Not always with natural parents	0.68		1.57	.008	.007
Not being a planned child		1.15		.327	.201
**Parental disorders**					
Mother physically ill		0.99		.970	.123
Father physically ill		1.48		.077	.108
Mother any chronic pain		1.22		.306	.019
Father any chronic pain	4.36		0.86	< .001	.001
Mother mental problems		1.25		.259	.022
Father mental problems		1.34		.215	.525
Mother alcohol problem		1.05		.870	.098
Father alcohol problem		1.36		.047	.921

^1^ Note: Displayed are p-values of the ordered logistic regression analyses for the main effect and the interaction between sex and the respective risk factor. ORs are reported separately for women and men if the interaction is significant. If it is non-significant, a common value for women and men is reported. Age, gender and country is always added as a confounder. No tests for other interactions (with age or country) reached significance, see text.

## Results

Out of the 38 bivariate associations that were tested, three reached significance: "Father had chronic pain" in women, "neglect" in men and "mother had mental disorder" in men (Table 3). All were larger than one indicating that they constitute risk factors rather than protective factors. In the adjusted analysis (ordered regression), no significant interactions were found between any of the risk factors and country or age. However, there were significant main effects and two interactions with gender. There was a decrease in headache with age (OR per decade = .84, p < .001). A squared term for age was tested but missed significance (p = .040). Women reported more headache than men (OR = 3.49, p < .001). There were no differences in headache frequency between Poland and Germany (OR = .81, p = .091). Hence, Table 4 shows only the interaction terms for gender, but ORs are controlled for the main effects of gender and age.

Generally, not many risk factors for headaches were identified. Most ORs were larger than one, but still small and insignificant. There were three exceptions. Neglect constituted a strong risk factor with an OR of 2.78 (p < .001, explained variance of Δ Pseudo R^2^ = 0.45%). The interaction effect for neglect*gender showed a p-value of .042. Because it was larger than our criterion alpha, it was not regarded. However, one could plausibly argue that the value was close to the nominal significance level and it would be incorrect to ignore it, particularly since it is known that the statistical power to detect interactions is usually low. Therefore, we examined it. The adjusted OR for women was 3.08, the one for men 2.66. Averaging over the two does not do much harm in this case. A second significant result was that paternal chronic pain was associated with more headaches in women, but not in men. This result was highly significant for women (adjusted OR = 4.00, p < .001), and clearly non-significant for men (p = .682, explained variance of Δ Pseudo R^2^ = 0.77%). Maternal chronic pain had no association to headaches, but again the interaction effect was close to becoming significant (p < .019). Therefore, we examined it as well. The adjusted OR was 1.91 for men, and 0.81 for women, both non-significant when tested individually against 1. A third significant interaction concerns the variable “Not always having grown up with natural parents”. It showed a significant interaction with gender, too. Women who assented this item reported less headaches (adjusted OR = .68, p = .052), men more (adjusted OR = 1.57, p = .065). Both effects themselves were not significant against 1, however the difference between women and men was significant (p = .008, explained variance of Δ Pseudo R^2^ = 0.36%). There were no differences in the rates of girls and boys who did not always grow up with a biological parent, about 20% for both. The significant bivariate effect for "mother having any mental problems" in men did not remain significant after adjusting for confounders.

To explore if the 16 childhood adversities which did not display any significant effect on headaches individually may do so if analyzed cumulatively, an unweigted sum score was calculated over the 16 risk factors. The score had a Cronbachs alpha of .72 and an odds ratio with headaches of 3.26. It was non-significant (p = .011) and showed an explained variance of Δ Pseudo R^2^ = 0.31%.

## Discussion

In sum, the present results indicate that for adult headaches, childhood adversities do not constitute strong risk factors. A total of 16 out of the 19 factors explored here showed non-significant associations as well as their sum score. Naturally, this is at least partly a consequence of the low significance level that was chosen. However, we set it low intentionally to avoid spurious results, considering that six statistical tests per risk factor sum up to 114 tests in total. On the other hand, such a lack of significant associations was surprising, because it partly stands against the results from other studies examining adult headaches and childhood adversities (e.g. [20]). We did not put much weight to the result regarding the cumulative score of the 16 individually non-significant risk factors due to two reasons: (1) It was non-significant even if close to the boarder. (2) It explained less variance than any of the three individually significant effects.

However, the three individual risk factors that were identified deserve attention. The most interesting result was the one for neglect. It turned out to be a relatively strong risk factor for headache, both for women and men, in Poland as well as in Germany. Neglect is a risk factor that has been explored rarely in research, even though it is one of the most frequent childhood adversities identified by the child protection services [21]. Some studies explored neglect and found mostly smaller effects than for other childhood adversities. Scott et al. [14] for example found a hazard ratio (HR) of 1.21 for neglect and headaches, while other childhood adversities such as physical and sexual abuse had higher HRs (e.g. HR_phys abuse_ = 1.64, HR_sex abuse_ = 1.73). The present indicator for childhood neglect was a scale combined from five items for physical and five for emotional neglect and not a single item—Scott et al. used a single item. Perhaps this contributes to the higher effect size.

The design of the present study does not allow to draw causal conclusions. It is clearly possible that the association between childhood neglect and adult headaches was generated by third variables, for example a certain answering style. Genetic effects were proven in the genesis of headaches [22–24], but it is hard to imagine that they may increase the risk for childhood neglect. About the mechanism how childhood neglect may increase the risk for adult headache we can only speculate. There are results from mice showing that neglected pups display different pattern of methylation than well cared ones (e.g. [25]). A more psychological view would be that a neglected child will probably realize at some age that her or his parents do not care as much as other parents do. But before such a realiziation, it may already develop feelings of being not worth to be loved, being guilty for something or even feels shame for the parents [26]. Such feelings would rather lead to a mechanism of internalizing negative emotions than to externalize them. Internalizing anger and suppression of feelings were shown to be risk factors for headaches [27, 28]. Poor ability to regulate negative emotions seems to generally be associated with chronic pain [29]. Other forms of abuse, e.g. sexual or physical abuse show similar immediate consequences [30], but here it may be easier for the children to reattribute the responsibility in later years to the adults. In this context, it would have been interesting to distinguish between physical and emotional neglect because we would rather expect the emotional neglect to be associated with headaches. However, we were not able to do so, even though we used standard questions like many other researches, too [17]. We think that more research would be necessary on childhood neglect as a risk factor for later mental and physical health.

The two interaction effects that were detected are more difficult to interpret. From the parents as a model hypothesis, it seems paradoxical that women from fathers with chronic pain have a higher risk of developing headaches in adulthood, but not men. A less pronounced but similar pattern was observed for men and maternal chronic pain. Hence, a different mechanism may act as a mediator in this case. We explored the hypothesis that girls would have had to care for fathers with chronic pain, in the sense of insecure attachment or more specifically parentification [31]. Hence, we tested whether paternal parentification was a mediator between paternal chronic pain and headache in the sense of Baron and Kenny [32]. This was not the case. A second hypothesis was that men would overlook their fathers’ pain because they did not care and consequently tended not to report it in our survey. This was also tested but found to be untrue: the rate of men and women reporting fathers’ pain was similar and the difference non-significant. So, the explanation for the effect probably lies elsewhere. Before such an effect can be interpreted in detail, we want to repeat the analysis on a different data set. In a new study, we would also ask for the location of the parents’ pain. Then, it would be possible to check whether offspring headache is associated with parental headache, while other pain locations in parents may possibly show different patterns.

Similarly problematic to interpret is the second interaction. About 20% of both girls and boys did not always grow up with their biological parents. For boys, this was associated with a higher risk for headache, for girls with a slightly lower one. One could say that because both effects were non-significant against one, it would be superfluous to interpret it at all, but this would not be fair insofar as the difference between girls and boys was significant according to our alpha level. There have been some comments in the literature that boys may be more sensitive than girls regarding various childhood adversities(e.g. [33, 34]), but this would still not explain the effect that partly growing up somewhere else other than with biological parents can have a positive effect for girls. Most likely, boys and girls were taken out of their families for different reasons, but unfortunately this was not assessed in our questionnaire. If we repeat this research using a different sample, we would add an open field to this question requesting the reason for not growing up with biological parents.

The present study has the following limitations. First, data were assessed retrospectively via an internet survey. It is unknown how much of a selection bias or of a memory bias is present. There is an ongoing critique on how valid such data are (e.g. [35, 36]), while other studies support the use of retrospective data from childhood [37, 38]. Second, headaches were analyzed as a primary response. Since some of these may have a fluctuating course, complaints by subjects who currently feel well but who have had serious phases of impairment are left out. Third, headaches were assessed using only a single item. There is no possibility to assess its reliability. Additionally, the answering categories may have had a different meaning for different subjects. Fourth, anxiety and depression were not considered, here, which both show associations to pain [39]. Fifth, ORs were chosen as an indicator for the relationships. It is known that ORs tend to overestimate associations under certain conditions [40].

Beside these limitations, the study has the advantage of relying on a relatively large sample from two different countries. The advantage of a multi-language survey is that in case of a congruence of effects, as was consistently the case in the present study, the likelihood that effects are mainly a result of the phrasing of the items decreases. However, when lining up the present study alongside previous research, the magnitude of the associations estimated here would surely fall into the lower rank of estimating the strength of the influence of childhood adversities on adult headaches. Even if many ORs here would not statistically differ from those estimated in some previous studies(e.g [14, 41]), the conclusion for current practice would differ: i.e. not to focus too strongly on childhood adversities when thinking about causes for adult headaches—other factors may be more important. Psychosocial difficulties and dysfunctional coping are important for patients with headache, but proximal probably more than distant ones [8].

## Conclusion

There is no doubt that childhood adversities as well as adult traumata show long term sequelae, visible in mental (e.g. [42]) and physical (e.g. [20]) aspects. But headaches do not seem to be a typical long term sequel of most childhood adversities, what is actually surprising. The by far most prevalent forms of headaches are tension type headaches and migraine. In both, psychological factors usually are assumed to constitute important roles in the genesis—at least in their chronic forms(e.g. [43, 44]). However, the sequelae of most childhood adversities do not seem to manifest as headache, with one important exception: childhood neglect. For future research, the conclusion of this study would be to pay more attention to neglect [45] and to keep possible gender interactions in mind.

## Abbreviations

OR: odds ratio
HR: hazard ratio
